# Association between Bone Turnover Markers, Leptin, and Nutritional Status in Girls with Adolescent Idiopathic Scoliosis (AIS)

**DOI:** 10.3390/nu12092657

**Published:** 2020-08-31

**Authors:** Edyta Matusik, Jacek Durmala, Magdalena Olszanecka-Glinianowicz, Jerzy Chudek, Pawel Matusik

**Affiliations:** 1 Department of Rehabilitation, Faculty of Health Sciences in Katowice, Medical University of Silesia, 40-752 Katowice, Poland; jdurmala@sum.edu.pl; 2 Health Promotion and Obesity Management Unit, Department of Pathophysiology, Faculty of Medical Sciences in Katowice, Medical University of Silesia, 40-752 Katowice, Poland; magolsza@gmail.com; 3 Department of Internal Medicine and Oncological Chemotherapy, Faculty of Medical Sciences in Katowice, Medical University of Silesia, 40-752 Katowice, Poland; jchudek@sum.edu.pl; 4 Department of Pediatrics, and Pediatric Endocrinology, Faculty of Medical Sciences in Katowice, Medical University of Silesia, 40-752 Katowice, Poland; endocrin@wp.pl

**Keywords:** adolescent idiopathic scoliosis, bone turnover markers, leptin, body composition

## Abstract

The link between scoliotic deformity and bone metabolism in adolescent idiopathic scoliosis (AIS) has not been well researched. Moreover, the data concerning the cross-talk between fat tissue content/hormonal activity and bone markers in this group of patients are lacking. The aim of the study was to assess whether there exists a significant relationship between the severity of AIS and bone turnover markers and leptin levels. The study group was consisted of 77 AIS girls, aged 14.7 ± 2.17 years. Scoliotic curve severity assessed by Cobb’s angle was categorized as mild (10–19°), moderate (20–39°), or severe (≥40°). Corrected height, weight, and waist and hip circumferences were measured and body mass index (BMI), corrected height Z-score, BMI Z-score, and waist/height ratio (WHtR) were calculated for the entire group. Body composition parameters: fat mass (FAT), fat-free mass (FFM), and predicted muscle mass (PMM) were determined using a bioelectrical impedance analyzer. Bone turnover markers (osteocalcin (OC) and amino terminal of collagen cross-links (NTx) and leptin levels were assessed in serum. Multiple regression analysis showed that, OC, NTx (negatively with *p* < 0.05), and leptin (positively with *p* < 0.01) were significantly associated with curve severity in AIS girls. Moreover, Cobb’s angle was positively correlated with W/HtR (*p* < 0.01) and FAT (*p* < 0.05). One-way analysis of variance (ANOVA) revealed significant differences in leptin (*p* < 0.05 vs. mild only), OC (*p* < 0.05 vs. mild and moderate), and W/HtR (*p* < 0.01 and *p* < 0.05 vs. mild and moderate, respectively) between the three AIS severity subgroups. OC was significantly lower in the severe AIS subgroup, while leptin and W/HtR were significantly higher. Significant correlations between leptin and anthropometrical parameters as BMI z-score and W/HtR were shown. Leptin level correlated also significantly with BMI z score (*p* < 0.001), W/HtR (*p* < 0.0001), and body composition parameters (*p* < 0.000001). Moreover, there was a significant negative correlation between NTx and leptin level (*p* < 0.05). Bone metabolism in AIS girls seems to be altered and significantly related to the scoliotic curve severity. Leptin may be a crucial link in the cross-talk between bone turnover and body composition in this group of patients. Further studies concerning interrelationship between nutritional status and bone metabolism in patients with AIS are warranted.

## 1. Introduction

Adolescent idiopathic scoliosis (AIS) is the most prevalent form of spinal deformity during the period of growth spurt and pubertal development. The most important clinical issue in the patients with AIS is the deformity progression. Pathogenesis of AIS probably has a multifactorial background [1,2,3,4,5,6] and is still the matter of discussion [7,8]. Abnormal growth pattern during puberty was considered as one of the possible etiological models of AIS pathogenesis. In the study conducted by Nicolopoulos et al., adolescent girls with AIS had longer lower extremities with taller both total and sedentary heights and these silhouette components were significantly different than in girls from the control group [5]. Similar findings were described in the group of 598 Chinese girls with IS [9]. Prepubertal girls with idiopathic scoliosis were significantly shorter and had shorter sedentary height and between-arms distance compared to the control group, in contrary to the period of puberty progression, the height, sedentary height, and between-arms distance were significantly greater in girls with IS [9]. Previous studies found that AIS is associated with low bone mineral density (BMD) and abnormal bone quality and strength [10,11,12,13,14], whilst the link between scoliotic deformity and bone metabolism in adolescent idiopathic scoliosis, (AIS) has not been well researched and the exact mechanisms and causes of the bone loss in AIS are not identified yet. Recent study assessing bone mechanical properties in AIS patients showed that osteopenia in this group of patients might be the result of abnormal regulation and modulation of bone metabolism and bone modeling/remodeling [14].

Bone turnover markers (BTMs) are a group of mainly protein or protein derivative particles which are released by osteoblasts or osteoclasts during bone remodeling process. BTMs can be used as prognostic factors for the risk of fractures in patients with osteoporosis and can be very important supplementary tool to the radiographic measures of bone mass (as DXA). BTMs are also very useful scientific tool reflecting changes in both bone physiology and different types of pathology [15]. The most commonly used in both clinical and scientific practice are osteocalcin (OC), reflecting bone formation by osteoblasts an N-terminal telopeptides of collagen type 1 (NTx), reflecting bone resorption by osteoclasts. Usefulness of OC, NTx, and OC/NTx ratio in pediatric population was previously studied by our group [16].

Leptin is a hormonal factor produced mainly by the adipocytes from leptin *(lep)* gene and is responsible mainly for satiety control within the hypothalamic–pituitary system and regulation of energy expenditure. It is also the crucial factor triggering the pubertal development especially in girls [17]. Leptin is also important factor influences both bone mineralization and growth [18,19,20]. Study designed by Qiu et al., showed that leptin level in girls with AIS was significantly related to the bone mineral density and was significantly lower than in controls group [3]. Leptin and its signaling pathway may be a candidate for the etiology of AIS. Leptin, together with the soluble leptin receptor (sOB-R), were shown to play an important role in the regulation of bone and energy metabolism in children. Leptin affects bone metabolism via central and peripheral ways. It modulates cortical bone formation by regulating the expression of several neuropeptides in hypothalamus and inducing sympathetic activation [18,19,21,22]. Based on recently published results, the other role of leptin in the development of scoliotic deformity may be related to the leptin resistance [23] or its lower bioavailability [24,25], which can lead to the development of important difference between growth velocity of spine compared to the extremities [1,4,6,26]. However, the data concerning the cross-talk between leptin level and bone markers in patients with AIS are lacking. Therefore, study showing the interrelationship between every scoliotic curve magnitude category (mild, moderate, and severe) in relation to the bone metabolism and leptin level is needed.

Accordingly, the aim of this study was to correlate the extent of scoliotic-curve severity with the bone turnover markers (osteocalcin (OC) and amino terminal of collagen cross-links (NTx) vs. leptin level and nutritional status in girls with AIS.

## 2. Materials and Methods

### 2.1. Studied Population

The study group consisted 77 girls with newly-diagnosed AIS, aged 14.7 ± 2.17 years. Patients were consecutively recruited during their first visit at the Scoliosis Clinic in our Department of Rehabilitation. The diagnosis was confirmed by both, clinical assessment and standard standing postero-anterior X-ray film of the spine with Cobb’s angle ≥10°. The exclusion criteria were as follows: any forms of prior treatment for scoliosis in anamnesis, mental retardation, endocrine disorders, congenital skeletal dysplasia or deformities, neuromuscular diseases, connective tissue abnormalities, or history of glucocorticoid therapy and osteoporotic fractures.

### 2.2. The Evaluation of the Scoliotic Curve Magnitude

Scoliotic curve magnitude was evaluated by measuring Cobb’s angle at the coronal plane of the whole spine on a standard X-ray film. When, the case of double or triple scoliotic curves occurred, the Cobb’s angle of the major curve was selected. Based on the conventional classification of Cobb’s angle [27], curve magnitude was categorized as mild (Cobb’s angle 10–19°), moderate (Cobb’s angle 20–39°), or severe (Cobb’s angle ≥40°).

### 2.3. Anthropometric Parameters

All the anthropometric measurements were performed at the first visit in the Clinic. Standing height was measured by a wall-mounted Harpender Stadiometer to the nearest 0.1 cm. Weight (in underwear) was measured with an electronic scale with readings accurate to 0.1 kg. The corrected height was derived for every AIS patient with Bjure’s formula (log *y* = 0.011 *x* − 0.177), where *y* is the loss of trunk height (cm) due to the deformed spine, and *x* is the greatest Cobb angle of the primary curve [28]. Body mass index (BMI) using the standard formula (kilograms per meter squared) was then calculated. Anthropometrical status (i.e., underweight, normal weight, overweight, and obesity) was diagnosed by BMI z-score after the adjustment of BMI value for age and sex. BMI *z*–score is expressed as a number of standard deviations (SD) from the value of the 50th percentile (median). BMI z-scores were derived using WHO AnthroPlus, version 1.0.4 (based on World Health Organization growth references) [29]. Waist circumferences were measured midway between the lower rib margin and the iliac crest in the standing position and waist to height ratio (W/HtR) was calculated.

### 2.4. Body Composition Analysis

Body composition analysis based on bioelectrical impedance analysis (BIA) with the use of segmental body composition analyzer (BC-418MA Tanita Europe BV, Hoofddorp, The Netherlands) was performed in all AIS group. Body composition parameters as fat mass (FAT), fat-free mass (FFM), predicted muscle mass (PMM), and total body water (TBW) were assessed (in kilograms (kg) or as percentage of body weight (%)) were recorded and analyzed.

### 2.5. Biochemical Analysis

Venous blood samples were drawn from antecubital vein in the morning in the supine position after the overnight fasting and collected in heparinized vacutainer tubes. After centrifugation at 1500× *g* at 4 °C for 5 min, plasma was collected and transferred in Eppendorf™ tubes, then immediately frozen and stored at −80 °C until analysis. Competitive-inhibition enzyme-linked immunosorbent assay (ELISA) was used to evaluate amino terminal collagen cross-links (NTx) in serum (Osteomark NTx Serum). Quantitative sandwich enzyme immunoassay technique was used for the measurement of osteocalcin (OC) (MicroVue Osteocalcin EIA kit, Quidel, San Diego, CA, USA) and leptin (TECOmedical AG, Swissach, Switzerland). All samples were tested in duplicate.

### 2.6. Ethical Considerations

The study was approved by the Ethics Committee of the Medical University of Silesia. All participants and/or their caregivers gave informed consent. Patient rights were also approved according to the Helsinki Declaration.

### 2.7. Statistical Analysis

The following variables were not normally distributed (assessed by Kolmogorov–Smirnov test) and were log transformed to achieve near-normal distributions: leptin, osteocalcin, and NTx. Multivariate regression analysis adjusted to age and Tanner stage was performed to identify the variables that influence the curve severity expressed as Cobb’s angle in the entire AIS population. Data are presented as the standardized regression coefficient (β) and adjusted r^2^. One–way analysis of variance (ANOVA) was used to analyze any significant difference among the three curve magnitude subgroups, i.e., mild, moderate, and severe. Correlations between continues parametrical (or log transformed) variables were based on linear Pearson’s correlation coefficient. All statistical analysis was made with the Statistica™ 12 PL software and *p* value less than 0.05 was considered statistically significant.

## 3. Results

Baseline characteristics and anthropometric measurements of all studied girls are reported in Table 1.

Multiple regression analysis using an age and Tanner stage adjustments was performed on the curve severity with respect to the bone markers, leptin, and anthropometrical data. Bone turnover markers (OC and NTx) and leptin were found to be significantly and independently associated with curve severity in the studied AIS girls. Bone turnover seems to be negatively associated with the curve severity, while leptin level has a positive relation to the deformity magnitude. Moreover, Cobb’s angle was positively correlated with W/HtR and FAT independently from age and Tanner stage. (Table 2). There was no significant relationships between curve magnitude and anthropometrical status defined by BMI z-score.

For the next stage of the analysis, the study group was further divided according to the curve magnitude i.e., mild (10–19°), moderate (20–39°), or severe (≥40°). The mean Cobb’s angles in the mild (*n* = 36), moderate (*n* = 30), and severe (*n* = 11) groups were 19.96 ± 7.92° and 52.36 ± 12.54°, respectively.

**Figure 1:**
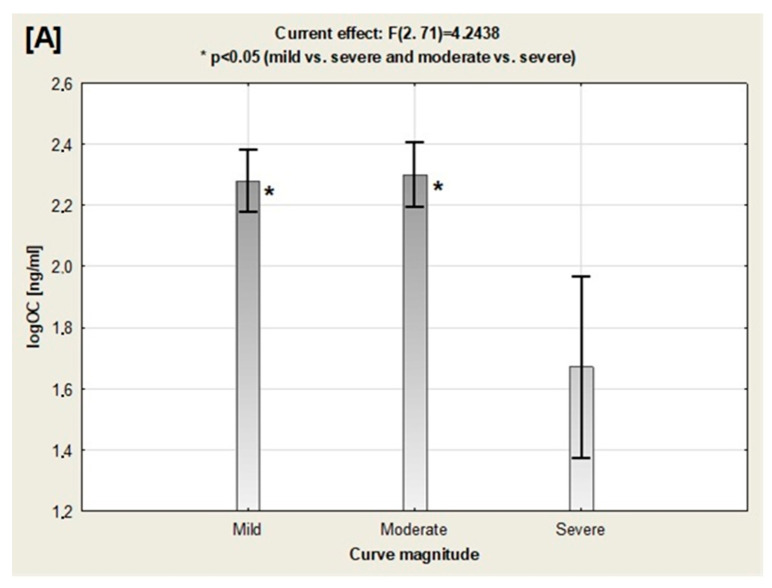

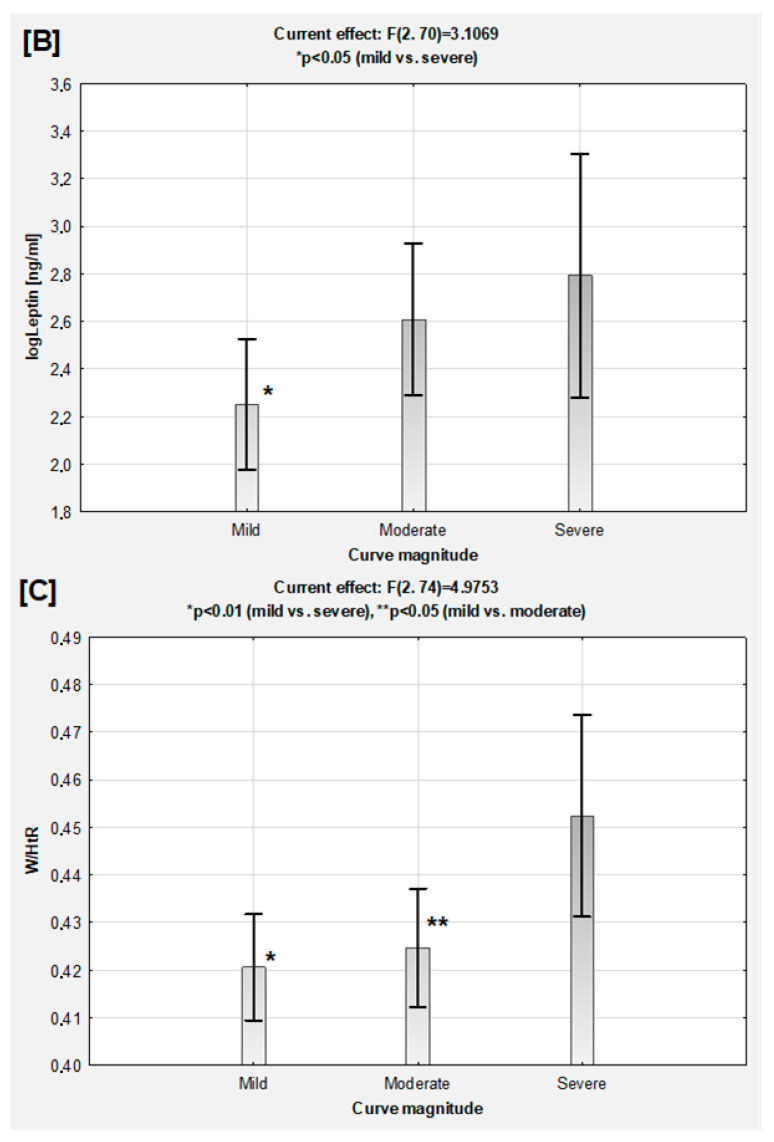
Comparison of osteocalcin (OC) (**A**), leptin (**B**), and waist to height ratio (W/HtR) (**C**), among the scoliotic curve severity subgroups by one-way analysis of variance (ANOVA).

One-way analysis of variance (ANOVA) revealed significant differences in leptin, osteocalcin, and W/HtR between the three scoliotic severity subgroups (Figure 1). Osteocalcin was significantly lower in the severe AIS subgroup than either in the mild and moderate subgroups (Figure 1A). Interestingly, leptin level (which is the marker of the adiposity) was significantly higher in the severe AIS girls vs. mild subgroup only (Figure 1B). Furthermore, adipose tissue distribution (evaluated by W/HtR calculation) was significantly higher in the severe AIS subgroup comparing to the both mild and moderate AIS subjects (Figure 1C). However, other anthropometrical parameters (BMI, BMI z-score, FAT, FFM, TBW, and PPM) were no significantly different between all three AIS subgroups (Table 3).

Significant correlations between leptin and anthropometrical parameters as BMI z-score and W/HtR were shown in mild and moderate AIS subgroups as in all studied population. Moreover, leptin level correlated significantly with body composition in the manner as expected (vs. FAT positively and vs. FFM, TBW, and PMM negatively). The similar trend of correlation was also shown in the severe subgroup, but the lack of significance was probably related to the small number of patients (Table 4.).

**Figure 2:**
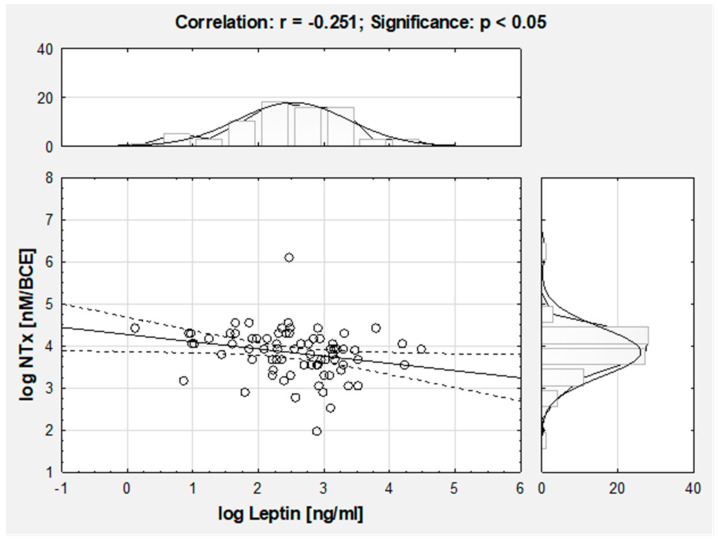
Correlation between amino-terminal collagen crosslinks (log NTx) and leptin (log Leptin) in the AIS girls.

Osteocalcin level showed significant negative relation to the BMI (r = −0.238, *p* < 0.05), but after adjustment to age by BMI z-score calculation this correlation was no longer significant. There were no other significant correlations between bone turnover markers vs. neither classical anthropometry and body composition parameters assessed by bioelectrical impedance. However, there was a significant negative correlation between NTx and leptin level (Figure 2).

## 4. Discussion

Presented cross-sectional study revealed the significant correlation between scoliotic curve magnitude and bone turnover markers and leptin level in AIS girls. Girls with severe AIS subgroup presented significantly more altered bone metabolism than mild and moderate AIS patients. Data coming from the present study confirmed also our previous findings [30], that the degree of spinal deformity was independently connected with type of the adipose tissue distribution and body composition (W/HtR and FAT) after age and Tanner stage adjustment. Interestingly, fat mass (FAT) and W/HtR, were correlated with Cobb’s angle in the same positive way as leptin level, whilst a significant correlation for bone turnover has the opposite negative relation.

Proper growth and stabilization of the skeletal system (especially the vertebral column) is strongly related to the accurate amount of both major body composition components i.e., adipose tissue and fat free mass. The routine assessment of nutritional status in developmental period is performed by correct measurement of height, weight, and body mass index (BMI) calculation. The obtained results cannot be interpreted as a raw data but have to be checked according to the percentile charts for sex and age. However, the set of anthropometrical measurements described above does not go into the details of the body composition components and adipose tissue distribution. When considering the majority of studies that have been performed in children with AIS, they show that the children’s BMI is lower than in the healthy controls [31,32,33]. Moreover, the results coming from longitudinal study published by Demerath et al. showed that changes in BMI percentile may not accurately reflect changes in adiposity, particularly in children and adolescents with low BMI [34]. Current gold standard for the body composition evaluation is dual-energy X-ray absorptiometry (DXA). However, more widespread use of DXA in children is limited mainly by its costs and exposure to X-ray radiation. Bioelectrical impedance analysis (BIA) is a noninvasive, relatively simple, readily accessible and quick, body composition assessment technique with a good correlation to DXA results [35,36]. Segmental BIA is well validated technic also in healthy children and adolescents and standardized centile FAT and FFM charts are now available [37]. In the present study body, FAT% assessed by BIA correlated significantly with Cobb’s angle, and there were also significant relations between all body composition parameters and leptin level. Present findings are complementary to our previous study results, which revealed that body composition parameters assessed by BIA were significantly related to the degree of spinal deformity and may be useful as a method of body composition assessment in patients with AIS [30]. Other study conducted by Ramirez et al. [38] showed that body composition parameters (assessed by BIA) were significantly lower in patients with AIS than in control group. However, these results may be related to the great variety of age (13–26 years) and high underweight prevalence (55.6%) in studied group. Moreover, studied group consisted girls only with the severe form of AIS (max. Cobb’s angle = 66°), the candidates for surgery. Unfortunately, the impact of body composition parameters on the scoliotic curve magnitude was not evaluated in this study [38].

In our study body composition parameters (FAT, FFM, TBW, and PPM) correlated significantly with leptin level. The same results was found in the very recent study conducted in the Chinese AIS population. Body composition parameters correlated with the same manner with both total leptin and free leptin index (FLI) in AIS group with the similar significance as in control group [24]. Study published by Lee et al. revealed that curve severity was inversely and independently associated with both axial and peripheral bone mineral density (BMD) [27]. These findings may explained our data showing negative correlation between curve magnitude and bone turnover markers.

Bone turnover markers used in our study included osteocalcin (OC), reflecting bone formation, and N-terminal telopeptides (NTx), reflecting bone resorption. In the present study both OC and NTx showed a significant inverse relationship to the scoliotic curve severity. Similar findings have recently been studied by Chen et al. who showed that OC level was significantly lower in the group with the severe AIS (Cobb’s angle ≥ 45°) compared to the mild subgroup. However, C-terminal telopeptides (CTx) used as a bone resorption marker in this study was insignificantly lower in mild and severe compared to control group [39]. Significant positive correlation of both OC and NTx with Cobb’s angle were found by Berglund et al. but in the group of patients with scoliosis related to fibrous dysplasia and McCune Albright Syndrome which are related to very high level of bone turnover [40]. We also found a significant negative OC correlation vs. BMI, but after age adjustment by the BMI z-score calculation the association was no longer significant. Similar relation between OC and BMI was found by Dubnov-Raz et al. in the group 160 of healthy adolescent girls [41]. The other study showed the inverse significant relation between OC and either adiposity (BMI and fat mass) and leptin level, but in the group of adolescent boys [42]. Currently serum OC levels are used to evaluate bone metabolism, as a bone formation marker. However, recently increasing data have emerged to support extra-skeletal effects of OC. Osteocalcin as a vitamin K related protein can be presented in serum in two major forms carboxylated OC (ccOC) and uncarboxylated OC (ucOC). The recent data suggest that ucOC may be involved in glucose homeostasis by both the direct influence on β-cells in pancreas and indirect by the adiponectin secretion which facilitate insulin sensitivity. The other possible extra-skeletal functions of OC are angiogenesis induction and fertility maintenance in males [18,19,43].

In our study leptin was negatively correlated with NTx in AIS girls. To our knowledge, such relation was not previously described in the literature. We can suspect that altered bone metabolism in the AIS patients seems to be related to the leptin activity pathway. Our data seem to confirmed the importance of leptin in the AIS pathogenesis in relation to the bone metabolism. A study conducted by Cheung et al. studied a population that included 598 girls with AIS and found that puberty was an important factor influencing abnormal growth pattern. In prepubertal girls (Tanner I) their height, sedentary height, and between-arms distance were significantly shorter compared to the control group; while with the puberty development (from Tanner II to IV), all these three parameters were significantly greater in girls with AIS [35]. However, bone turnover markers and leptin were not included to this analysis in reflect to the scoliotic curve severity. In our study, bone turnover markers and leptin were related to the curve severity independently form age and pubertal development (expressed as Tanner stage).

Waist-to-height ratio (WHtR) is nowadays intensively studied as a relatively simple anthropometric marker of fat tissue distribution with a good correlation with visceral fat tissue and its metabolic comorbidities. Moreover, recently published studies revealed WHtR as better marker than the waist/hip ratio (WHR) visceral obesity and metabolic syndrome components prognosis [44,45,46]. WHtR seems to be another relatively simple to measure and promising parameter for adipose tissue distribution in the pediatric population with AIS. In our present study, this parameter was significantly related to the scoliosis severity and leptin level. However, some technical issues have to be taken into account, especially in children with low BMI z-score and severe scoliotic deformity in lumbar spine. Correct measurement of waist circumference may be technically very difficult in that group pf patients. Therefore, the usage of WHtR as a potentially pragmatic parameter in patients with AIS needs further investigations.

The major limitations of our study are the lack of the possibility to evaluate the bone mineral density and a relatively low number of the severe curve magnitude patients. Nevertheless, the obtained differences concerning the bone turnover markers and leptin remained statistically significant. Furthermore, to our knowledge, there are no other publications concerning the relationship between bone turnover markers and leptin in AIS patients. Further research in that area of adolescent idiopathic scoliosis is warranted, especially in the subgroup of patient with severe form of AIS.

## 5. Conclusions

Bone metabolism in AIS girls seems to be altered and significantly related to the scoliotic curve severity. Leptin may be a crucial link in the cross-talk between bone turnover and body composition in this group of patients. The useful tools for anthropometrical analysis in that topic seem to be waist to height ratio (WHtR) and body composition based on bioelectrical impedance analysis (BIA). Further investigations concerning crosstalk between bone and adipose tissue especially in the relation to the curve magnitude in patients with adolescent idiopathic scoliosis are warranted.

## Figures and Tables

**Table 1:** Basal characteristics of the total studied Adolescent Idiopathic Scoliosis (AIS) population.

**N**	77
**Age (years)**	14.7 ± 2.17
**Corrected height (cm)**	166.55 ± 9.48
**Corrected height Z score (SD)**	0.93 ± 0.99
**Weight (kg)**	51.28 ± 9.62
**BMI (kg/m^2^)**	18.38 ± 2.56
**BMI Z score (SD)**	−0.70 ± 1.03
**W/HtR**	0.43 ± 0.04
**FAT (%)**	21.47 ± 5.56
**FFM (%)**	77.69 ± 9.03
**PMM (%)**	74.55 ± 5.73
**TBW (%)**	56.91 ± 6.34
**Cobb’s angle (°)**	25.21 ± 15.32

Data are expressed as mean ± standard deviation. Abbreviations: BMI—body mass index, FAT—fat mass, FFM—fat free mass, PMM—predicted muscle mass, TBW—total body water, and W/HtR—waist height ratio.

**Table 2:** Multivariate regression analysis of the variables influencing the curve magnitude of studied population (adjusted to age and Tanner stage).

Variable	Coefficient β	Cobb’s Angle	Adjusted R^2^
**Log Leptin (ng/mL)**	0.243	*p* < 0.05	0.062
**Log OC (ng/mL)**	−0.260	*p* < 0.05	0.082
**Log NTx (nM/BCE)**	−0.381	*p* < 0.01	0.109
**W/HtR**	0.314	*p* < 0.01	0.075
**FAT (%)**	0.268	*p* < 0.05	0.083

Abbreviations: FAT—fat mass, NTx—amino-terminal collagen crosslinks, OC—osteocalcin, and W/HtR—waist height ratio.

**Table 3:** BMI, BMI z-score, and body composition parameters in all AIS subgroups.

	Mild (*n* = 36) Mean ± SD	Moderate (*n* = 30) Mean ± SD	Severe (*n* = 11) Mean ± SD	Significance (ANOVA)
**BMI (kg/m^2^)**	18.13 ± 1.74	18.30 ± 2.82	19.08 ± 2.30	NS
**BMI z-score (SD)**	−0.74 ± 0.77	−0.81 ± 1.12	−0.44 ± 1.54	NS
**FAT (%)**	20.76 ± 4.82	21.47 ± 5.39	24.78 ± 7.54	NS
**FFM (%)**	79.24 ± 4.90	76.33 ± 12.71	75.24 ± 7.57	NS
**TBW (%)**	58.01 ± 3.58	55.99 ± 8.81	55.09 ± 5.54	NS
**PMM (%)**	74.96 ± 5.62	74.87 ± 5.37	71.43 ± 6.99	NS

Abbreviations: BMI—body mass index, FAT—fat mass, FFM—fat free mass, PMM—predicted muscle mass, and TBW—total body water.

**Table 4:** Correlations between leptin level and anthropometrical variables in the studied AIS subgroups.

Log Leptin [ng/mL]				
Pearson’s Correlation				
	Mild (*n* = 36)	Moderate (*n* = 30)	Severe (*n* = 11)	Total (*n* = 77)
**BMI z-score (SD)**	0.374 *	0.445 *	0.421 (*p* = 0.197)	0.372 ***
**W/HtR**	0.417 *	0.570 **	0.551 (*p* = 0.079)	0.487 ^
**FAT (%)**	0.694 ***	0.540 **	0.558 (*p* = 0.075)	0.583 ^
**FFM (%)**	−0.691 ***	−0.537 **	−0.551 (*p* = 0.073)	−0.581 ^^
**TBW (%)**	−0.694 ***	−0.532 **	−0.564 (*p* = 0.071)	−0.581 ^^
**PMM(%)**	−0.641 ***	−0.551 **	−0.556 (*p* = 0.076)	−0.570 ^^

Abbreviations: BMI—body mass index, FAT—fat mass, FFM—fat free mass, PMM—predicted muscle mass, TBW—total body water, and W/HtR—waist to height ratio. * *p* < 0.05; ** *p* < 0.01; *** *p* < 0.001; ^ *p* < 0.0001; ^^ *p* < 0.000001.
